# Eggs and Cardiovascular Disease Risk: An Update of Recent Evidence

**DOI:** 10.1007/s11883-023-01109-y

**Published:** 2023-05-23

**Authors:** Sharayah Carter, Elizabeth S. Connole, Alison M. Hill, Jonathan D. Buckley, Alison M. Coates

**Affiliations:** 1 Alliance for Research in Exercise, Nutrition and Activity (ARENA), Allied Health & Human Performance, University of South Australia, GPO Box 2471, Adelaide, 5001 Australia; 2 Alliance for Research in Exercise, Nutrition and Activity (ARENA), Clinical and Health Sciences, University of South Australia, Adelaide, Australia

**Keywords:** Egg consumption, Cardiovascular disease, Mortality, Stroke, Heart disease, Lipids, Blood pressure, Hypertension

## Abstract

**Purpose of Review:**

This review summarizes recent evidence published since a previous review in 2018 on the association between egg consumption and risk of cardiovascular disease (CVD) mortality, CVD incidence, and CVD risk factors.

**Recent Findings:**

No recent randomized controlled trials were identified. Evidence from observational studies is mixed, with studies reporting either an increased risk or no association of highest egg consumption with CVD mortality, and a similar spread of increased risk, decreased risk, or no association between egg intake and total CVD incidence. Most studies reported a reduced risk or no association between egg consumption and CVD risk factors. Included studies reported low and high egg intake as between 0 and 1.9 eggs/week and 2 and ≥14 eggs/week, respectively. Ethnicity may influence the risk of CVD with egg consumption, likely due to differences in how eggs are consumed in the diet rather than eggs themselves.

**Summary:**

Recent findings are inconsistent regarding the possible relationship between egg consumption and CVD mortality and morbidity. Dietary guidance should focus on improving the overall quality of the diet to promote cardiovascular health.

**Supplementary Information:**

The online version contains supplementary material available at 10.1007/s11883-023-01109-y.

## Introduction

Cardiovascular disease (CVD) is the leading cause of mortality globally [1]. Early studies, which indicated that elevated serum cholesterol was associated with an increased risk of heart disease [2, 3], led to the American Heart Association (AHA) recommending limiting dietary cholesterol to less than 300 mg/day with specific recommendations to restrict egg consumption, which are high in cholesterol, to a maximum of three eggs per week [4]. A later analysis from the Framingham study found no association between egg intake and blood cholesterol or heart disease [5], and in 2002, the AHA removed its advice to limit egg intake while retaining its recommendation to consume less than 300 mg/day of dietary cholesterol [6]. During this time, findings surfaced indicating that increased intake of dietary cholesterol was associated with decreased synthesis of endogenous cholesterol [7], and in 2013, the AHA announced that “there is insufficient evidence to determine whether lowering dietary cholesterol reduces low-density lipoproteins (LDL) cholesterol” [8]. As a result, the 2015–2020 Dietary Guidelines for Americans removed the recommendation of setting a limit to the maximum intake of 300 mg/day of cholesterol. However, the controversy around the impact of consuming foods high in cholesterol, including eggs, on CVD risk remains.

A possible explanation for the controversy is that foods high in cholesterol are also typically high in saturated fat [9], which is well documented to increase LDL cholesterol and CVD risk [10]. Thus, it is difficult to determine the independent effects of dietary cholesterol on the blood lipid profile. Based on nutrient profiles from Australian food databases, eggs are high in cholesterol but low in saturated fat, with the average large whole egg (50 g) containing 244 mg of cholesterol but only 1.2 g of saturated fat [11]. However, eggs, like any individual food, are not consumed in isolation, but as part of an overall diet, which can influence total cholesterol and saturated fat intake. For example, in the American diet, intakes of cholesterol and saturated fat increase in parallel, which may be due to eggs being frequently consumed with bacon or sausage which are high in saturated fat [12]. Previous reviews considering egg consumption and blood lipids have reported contradictory findings, with a 2018 review of randomized controlled trials (RCT) finding that consuming eggs does not adversely affect the blood lipid profile [13]. However, in contrast, a subsequent meta-analysis of RCTs reported a positive relationship between changes in dietary cholesterol (all sources, including eggs) and changes in LDL cholesterol, after controlling for saturated fat [14], and so, the relationship remains unclear.

When the relationship between egg consumption and CVD risk has been reviewed, there have also been mixed findings. The majority of systematic reviews and meta-analyses observed no association between egg consumption and CVD risk [15–20], but a small number of studies identified an increased risk [21, 22], particularly in people with diabetes [17, 18, 23]. These inconsistencies in study findings continue to fuel the controversy around the impact of egg consumption on CVD risk.

This review aims to update current evidence identified from a systematic search and narrative review of observational studies and RCTs from 2019 onwards to explore the association between egg consumption and CVD risk, specifically, CVD mortality, incidence, and risk factors in adults. In addition, confounding factors that may play a role in the associations found will be discussed.

## Methods

### Search Strategy

An electronic search of PubMed, Web of Science, and Embase was conducted from January 1, 2019, to September 14, 2022, to identify the potentially eligible studies recent studies with the following search strategy: “[(egg OR eggs) AND (cardiovascular diseases OR cardiovascular OR coronary heart disease OR CHD OR CVD OR stroke OR myocardial infarction OR ischemic heart disease OR ischemic stroke OR hemorrhagic stroke) AND (randomized control trial OR RCT OR cohort OR prospective OR longitudinal OR follow-up OR case-cohort OR nested case control).” Studies were selected if they met the following inclusion criteria: (i) they were conducted on human adults; (ii) evaluated associations between egg intake and risk of CVD (fatal and nonfatal); (iii) evaluated associations between egg intake and CVD risk factors (e.g., hypertension, blood lipid profile, body fat mass). All references were evaluated by two independent reviewers (all by ESC, half each by SC and AMC) with an independent reviewer (either SC or AMC) resolving any disagreement.

### Data Extraction

Data were extracted by two independent reviewers (ESC and SC) from each identified study using a standardized extraction form. The following information was collected: (i) author names; (ii) year of publication; (iii) study cohort name and country; (iv) sample size, sex, and age (mean or range) of participants; (v) study aim and design; (vi) follow-up period; (vii) exposure type/dose/frequency; (viii) CVD outcome measures; (ix) CVD results; (x) covariates used in adjustments.

## Results

**Fig. 1:**
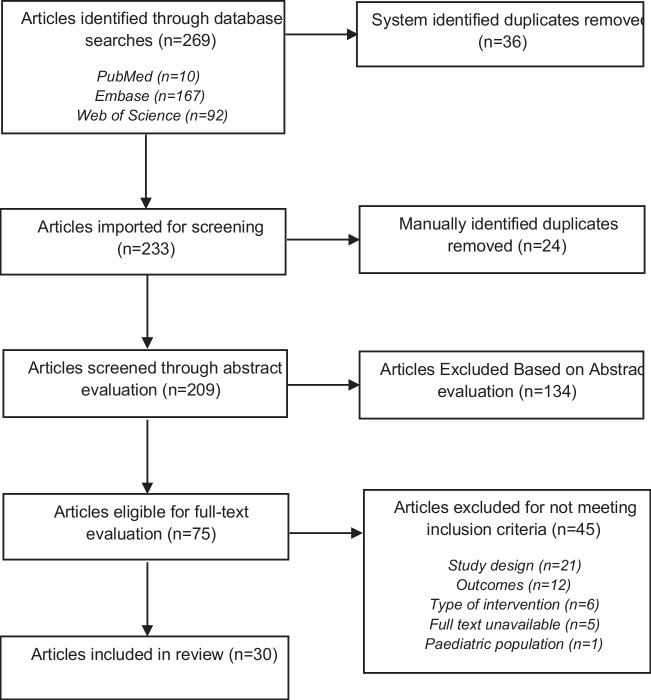
Flow Diagram of search strategy and included articles

Out of 269 references published during the four-year search period, 209 abstracts were screened, 75 were eligible for full-text screening, and 30 studies (35 data sets) were identified to review (all observational, no RCTs) (See Fig. 1). From the 30 studies, 13 were conducted with data from populations living in the USA [24, 25••, 26–29, 30•, 31–34, 35•, 36], eight from China [15, 35•, 37, 38••, 39–42], eight from Europe [43–50], one from Iran [51], and one was a multinational cohort study [52•].

The majority (> 50%) of studies controlled for age, gender, education, smoking status, alcohol consumption, physical activity, energy intake, BMI, hypertension, and diabetes. Two studies (7%) controlled for saturated fat intake [25••, 30•], eleven studies (37%) controlled for vegetable intake [28, 29, 36, 38••, 39, 41, 42, 48–50, 52•], six studies (20%) controlled for meat intake [26, 38••, 39, 41, 50, 52•] (two (6%) for processed meat [26, 50]), three (10%) for Mediterranean diet score [28, 44, 46], three (10%) for dietary approaches to stop hypertension (DASH) score [31–33], and six (20%) for diet quality [24, 26, 34, 35•, 37, 51]. Three (10%) studies which were across multiple countries controlled for center or region but not ethnicity [45, 48, 52•].

### Eggs and CVD Mortality

In the past 4 years, 11 observational studies [15, 24, 27, 29, 30•, 34, 35•, 43, 44, 46, 52•] have examined the associations between eggs and CVD mortality (See Supplementary Table 1). Of these, six publications reported on CVD mortality [24, 29, 30•, 34, 43, 46], and all except one [43] reported a higher risk of CVD mortality with the highest egg intake vs. the lowest egg intake (hazard ratio (HR) ranging from 1.14–1.75). Four out of five studies reporting a higher risk were from the USA [24, 29, 30•, 34] with the remaining study from Italy [46]. One of these five studies [30•] was a substitution study that reported a reduced risk of CVD mortality (total, heart disease, and stroke [males only]) when 3% of energy from plant protein was substituted for egg protein (HR range 0.72–0.76). In one study [24], the significant association was lost after adjusting for dietary cholesterol, and no association was found for egg white consumption. Five publications [15, 27, 35•, 44, 52•] reported no association between eggs and CVD mortality (one reported on heart disease mortality only [27]). One study [35•], after grouping cardiometabolic subtypes (coronary heart disease (CHD), stroke, and diabetes) together, found that the highest egg intake in the White American cohort was associated with higher cardiometabolic mortality, reduced risk was found with moderate egg intake in the Chinese cohort, and no association was found in the Black American cohort.

### Eggs and CVD Incidence (Non-Fatal CVD)

Fifteen observational studies reported on egg consumption and CVD incidence (non-fatal CVD) in the last 4 years [25••, 26, 28, 31–34, 38••, 39, 45, 48–51, 52•] (See Supplementary Table 1). Eight reported on total CVD incidence [25••, 26, 28, 34, 38••, 39, 51, 52•] with four studies finding an increased incidence (HR ranging from 1.06–1.39) with the highest (≥ 7 eggs/week) vs. lowest egg intake (< 1 egg/week) [34], high (> 6 eggs/week) and low intake (< 3 eggs/week) [38••], or with a 0.5 egg/day continuous intake [25••], noting that in this last study, significance was lost after adjusting for dietary cholesterol. The fourth study was a substitution study that reported that substituting eggs with fish, nuts, legumes, or whole grains was associated with 2–3% lower relative risks for incident CVD when the substitution amount was one serving per week and 15–21% lower relative risks when the substitution amount was one serving per day [26]. Two studies found a decreased incidence (HR ranging from 0.78–0.89) [39, 52•] (data from the PURE study [52•]), and three found no association [28, 51, 52•] (data from the ONTARGET/TRANSCEND study [52•]).

Five studies reported on egg intake and stroke incidence with three finding no association [49, 51, 52•] and two finding an increased risk (HR ranging from 1.07–1.34). One study reported an increased risk of total stroke and ischemic stroke with high and low egg intake (> 6 and < 3 eggs/week for total stroke, and > 6 and < 1 egg/week for ischemic stroke) and an increased risk of hemorrhagic stroke with low egg intake (< 3 eggs/week) [38••]. The other study found an increased risk of 7% for total stroke and 25% for hemorrhagic stroke with 20 g egg/day continuous intake (total: 95% CI 1.01–1.14, *P* trend = 0.031, hemorrhagic: 95% CI 1.09–1.43, *P* trend = 0.002) but no association for ischemic stroke [45].

Individual studies investigating heart disease reported on the incidence of ischemic heart disease, coronary heart disease, myocardial infarction, or venous thromboembolism. Two studies reported on ischemic heart disease, one reporting an increased association with highest (≥ 7 eggs/week) vs. lowest egg intake (< 1 egg/week) [34] and the other showing a 7% decreased risk for continuous 20 g egg/day intake (95% CI 0.88–0.99, *P* trend = 0.023) [48]. Coronary heart disease incidence was reported in three studies, two finding no association [33, 51] except in a sub-analysis where an increased risk of 30% (95% CI 1.03–1.56) was found in the older cohort [33], and one finding an increased risk with highest (> 6 eggs/week) vs. moderate egg consumption (3 < 6 eggs/week) [38••]. Three studies reported on myocardial infarction incidence, one reported an 11–13% increased risk [32], one found no association [51], and one study with two data sets found a 17% reduced risk (data from the PURE study) and no association (data from the ONTARGET/TRANSCEND study) [52•]. No association was found for the incidence of heart disease [52•] or the incidence of venous thromboembolism [50].

### Eggs and CVD Risk Factors

Nine observational studies [15, 36, 37, 40, 42, 47, 49, 52•, 53] were identified in the last 4 years that examined the associations between egg consumption and CVD risk factors including lipid and lipoprotein concentrations, blood pressure (including the presence of hypertension), and adiposity (See Supplementary Table 1). Four publications examined the association between eggs and lipid profile [15, 36, 37, 52•]. Two studies found no association [36, 52•], one study found a decrease in lipid profile (with no association for high-density lipoprotein (HDL) cholesterol) [15], and one study found an increase in total cholesterol (TC) and LDL cholesterol but a decrease in triglycerides (TG) and an increase in HDL cholesterol [37]. One study [40] reported on the association between eggs and lipoprotein particle concentrations and found reduced risk [40].

Four studies examined eggs and blood pressure [15, 36, 49, 52•]. Two studies found reduced systolic blood pressure [15, 52•] (data set from the PURE study [52•]), and three studies found reduced diastolic blood pressure [15, 49, 52•] (data set from the PURE study [52•]) with the highest egg intake (> 45 g/day or ≥ 7 eggs/week). In contrast, one study reported increased systolic and diastolic blood pressure (data set from ONTARGET/TRANSCEND study [52•]). One study found no association between eggs and systolic blood pressure [49], and one study found no association for both diastolic and systolic blood pressure [36]. Two studies reported on the association between eggs and hypertension [42, 47]. No association was found for the highest (≥ 7 eggs/week) vs. the lowest (< 1 egg/week) egg intake, but an increased risk was found for lower egg intakes (2–6.9 eggs/week) [47]. A decreased risk was noted in a substitution study where eggs were substituted for meat [42].

Three studies described the associations between eggs and adiposity (BMI, body fat, waist circumference) [15, 36, 41]. One study [41] completed analysis by sex and found a decreased risk of adiposity for females with the highest (approximately 50 g/day) vs. lowest egg intake (0 g/day) but no association for males, and two studies found no association with eggs and waist circumference [15, 36].

### Ethnicity

The majority of research included in this review was from cohort data collected in three geographical regions, with population ethnicity influencing the direction of associations between egg consumption and CVD outcomes. Thirteen publications were from studies conducted in populations residing in the USA [24, 25••, 26–29, 30•, 31–34, 35•, 36]; twelve studies reported on eggs and non-fatal and fatal CVD [24, 25••, 26–29, 30•, 31–34, 35•] and one on risk factors [36], with seven identifying an increased risk [24, 25••, 26, 29, 30•, 31, 34], five indicating no association [27, 28, 32, 33, 36] and one indicating an increased risk (White Americans) or no association (Black Americans) [35•]. Eight studies used data from cohorts residing in China [15, 35•, 37, 38••, 39–42] with four studies reporting on eggs and non-fatal and fatal CVD [15, 35•, 38••, 39] and five reporting on eggs and CVD risk factors [15, 37, 40–42]. In contrast to the US findings, only one of these studies reported an increased risk [38••], six showed reduced risk [35•, 37, 39–42], and one found no association [15]. Eight studies used cohort data from Europe reporting on eggs and non-fatal and fatal CVD as well as CVD risk factors [43–50]. Three confirmed an increased risk [45–47], one found a reduced risk [43], and four found no association [44, 48–50]. One study from Iran found no association with non-fatal CVD [51]. A final study including cohort data from 21 countries also found no association between fatal CVD and CVD risk factors [52•].

### Subgroups

Eleven studies conducted subgroup analyses on eggs and CVD mortality, incidence, and/or risk factors [25••, 31, 32, 34, 38••, 39, 46–49, 52•]. Six studies evaluated sex differences: six for men [25••, 31, 38••, 39, 48, 49] and four for women [31, 38••, 39, 48]. All but two studies found no association or no difference to the overall study outcomes. One study, which reported no association in the total cohort, found a reduced risk of IHD in males [48], and another study that reported a reduced risk of total CVD and hypertension in the total cohort found no association for males but confirmed reduced risk for females [39]. Eight studies assessed BMI in a sub-analysis [31, 32, 34, 38••, 39, 46–48] with the majority (seven studies) reporting no association or no difference to the overall study findings. One study reported an increased risk of MI incidence for people with a BMI over 25 kg/m^2^ but found no association in the total cohort [32]. Nine completed sub-analyses for type 2 diabetes, and all but two found no association [25••, 31, 32, 34, 38••, 46–48]. The two studies [31] reporting an increased risk of CVD incidence in persons with type 2 diabetes (one specific to ischemic stroke [31]) also reported increased risk in their overall findings.

## Discussion

Eggs are a highly nutritious food; they offer a complete source of protein, containing all essential amino acids and a complement of vitamins and minerals [54]. However, the high cholesterol content in eggs makes them a food of concern, which has led to a plethora of studies over the last few decades investigating egg consumption and the risk of CVD.

In this narrative review (with studies identified by a systematic search), we summarized recent evidence from studies published from 2019 to September 2022 with regard to the impact of egg consumption on CVD risk. The studies were all observational, and the findings were mixed. For CVD mortality, an equal number of studies reported an increased risk or no association with the highest egg intake. This was similar to earlier reviews which also found mixed results. A meta-analysis of 39 observational studies including nearly 2 million individuals found no association between the highest intake of eggs and CVD mortality [55], and similar findings were presented in another meta-analysis of 24 observational studies of over 11 million individuals that found no association between highest intake of eggs and CVD mortality [56]. However, contrasting findings were reported in a meta-analysis of 19 observational studies that found a nonlinear dose–response association between egg consumption and CVD mortality, although the certainty of the evidence for these observations was rated as very low [57]. In this latter review, the majority (80%) of studies reporting an increased risk were studied from US populations. Similarly, in a meta-analysis which found no association in the total population, an increased risk was reported in a sub-analysis by ethnicity in the American population [56].

It is difficult to study food in isolation without considering the effect of foods consumed in the whole diet and the nutrients they collectively contribute. In Western populations, eggs are typically consumed with meat (often processed) which is high in saturated fat, while in Asian cultures, eggs are frequently consumed in meals with vegetables [27]. It is also possible that Western populations consume more cholesterol from other sources (e.g., red meat, full-fat dairy, and discretionary foods), as compared to people from Asian cultures [12, 58, 59]. In the American diet for example, due to dietary patterns, dietary cholesterol and saturated fat increase in parallel, e.g., eggs with bacon and sausage [12]. Cooking methods are also different, with higher temperatures and longer cooking time leading to oxidative damage to vitamins [60]. Furthermore, while nutrition databases have an average for the cholesterol content of eggs, this commonly differs by country and can be influenced by multiple factors including feed composition [61–63], housing systems (free range vs. caged) [64], and age of the laying hens [65]. These factors may contribute to the observed country differences. Very few studies in this review controlled for saturated fat; no studies have considered cooking methods, whether eggs were from free-range or caged chickens, or the feed they were provided; and only 37% controlled for vegetable intake, 20% controlled for red meat, and 6% controlled for processed meat which may confound associations.

Regarding CVD incidence (non-fatal), the findings of this review were also mixed. Almost an equal spread of increase, decrease, and no association between egg intake and total CVD incidence was found. Of the eight studies reporting an increased risk of a CVD event, six (75%) were from the USA, and in two of these studies, the populations were majority (92%) male. Therefore, similar to the mortality findings, there is a potential effect of ethnicity, sex, and dietary patterns. Although not consistently so, studies reporting increased incidence of CVD were often from older cohorts or studies with longer duration and hence an aging cohort, both of which would predispose to an increased risk of CVD incidence. Similar mixed findings have been reported in earlier review studies. A meta-analysis of 17 observational studies found no association with CHD or stroke and an increased risk of heart failure with high compared to low egg consumption [16]. In a meta-analysis of nine observation studies, eating one egg daily was not associated with an increased risk of ischemic heart disease (IHD) and was associated with a small reduction in stroke [15]. Similarly, a meta-analysis of 21 observational studies also found no association between egg consumption and CVD risk and found beneficial effects toward stroke risk [66]. An umbrella review of seven systematic reviews and 15 meta-analyses concluded that increased egg consumption is not associated with CVD risk in the general population [67].

In this review, most studies assessing egg consumption and CVD risk factors found a reduced risk or no association. Interestingly, the majority (56%) of studies were from Chinese cohorts, and only one study was from the USA. However, due to the exploratory nature of observational studies, these studies are unable to establish a causal relationship between egg intake and CVD risk factors. Randomized controlled trials are required to provide evidence of causation, and as there were no RCTs during the defined period of this review, our discussion will focus on review studies of earlier RCTs that examined eggs and CVD risk factors. A systematic review of 23 RCTs found nonsignificant effects of increasing the consumption of eggs on risk markers for CVD [13]. In a meta-analysis of 28 RCTs, high egg consumption increased total cholesterol, LDL, and HDL cholesterol, but not LDL-to-HDL or TC-to-HDL ratios or triglycerides compared with low egg control diets [68]. In another meta-analysis of eight RCTs comparing greater than four eggs/week to less than 4 eggs/week, egg consumption was not associated with differences in blood lipid profile or blood pressure [69] and in two meta-analyses looking at the effects of different food groups on hypertension, regular egg consumption was associated with a lower risk of hypertension [70, 71].

In summary, recent data on the associations between egg consumption and risk of CVD mortality, incidence, and risk factors is mixed. With observational data, it is difficult to assess the relationship of any individual food independently of a dietary pattern. The risk associations reported in the reviewed observational studies are likely to be attributed to the dietary pattern accompanying high egg intake (e.g., eating eggs with bacon and sausage or as part of a meal with vegetables) and/or other risk factors present in people with high egg consumption. For example, in Asian cultures, egg consumption is positively associated with socioeconomic status and physical activity, inversely associated with smoking, and generally correlated with other aspects of a healthy dietary pattern (e.g., higher intake of fiber, vegetables, and fruit) [15]. In the USA, egg consumption is correlated with low physical activity, smoking, and dietary patterns high in saturated fat (e.g., full-fat dairy, red meat, and processed meat) [72]. Very few of the studies reviewed adjusted analyses for dietary confounders like meat, processed meat, vegetables, and saturated fat, and this could have impacted outcomes. Therefore, the suggestion that egg consumption by itself promotes the risk and development of CVD is questionable compared with the overall complexity of the dietary pattern, physical activity, and genetic predisposition. There is, however, evidence suggesting higher egg consumption could be associated with a higher risk of CVD in people with diabetes [17, 18, 73], but not all studies confirm these findings, specifically after adjusting for background diet [74]; further studies are needed. The best evidence for CVD prevention supports adopting a change in overall dietary pattern [75], and therefore, dietary guidance to reduce CVD risk should focus on implementing a healthy dietary pattern rather than removing a single food, such as eggs.

## Supplementary Information

Below is the link to the electronic supplementary material.

Supplementary file1 (DOCX 59 KB)
