# Supplementary material for: Eggs and Cardiovascular Disease Risk: An Update of Recent Evidence
Source: Curr Atheroscler Rep. 2023 May 23;25(7):373–80. doi: 10.1007/s11883-023-01109-y (PMC10285014; doi:10.1007/s11883-023-01109-y)
Supplement: Supplementary file 1 — Supplementary file1 (DOCX 59 KB) [file 11883_2023_1109_MOESM1_ESM.docx]

**Supplementary Table 1. Characteristics and outcomes of studies assessing associations of egg consumption CVD risk**

| **Reference, country, data source, study design** | **Population characteristics,**  **follow up (years), sample size (n)** | **Egg amount,**  **exposure: categorical, exposure: continuous** | **Study outcomes** | **Risk profile** | **Model for analysis** |
| --- | --- | --- | --- | --- | --- |
| **Egg Consumption and CVD Mortality** | | | | | |
| Chen et al. 2021 (34), United States, Women’s Health Initiative, Prospective Cohort | Post-menopausal women in United States aged 50-79 years  17.8 years  *n* = 96831 | 1 egg = 1 large whole egg  *n* eggs/week: <1, 1<2, 2<3, 3<7, ≥7  0.5 egg/day increment | Highest intake v lowest intake: HR 1.23, 95% CI 1.10, 1.39, *P*trend=0.001  Second highest intake v lowest intake: HR 1.11, 95% CI 1.02, 1.20, *Ptrend*=0.001  Continuous per 0.5 egg/week: HR 1.10, 95% CI 1.05-1.15 | ↑ | Age, region, ethnicity, socioeconomic status (education, family income, health insurance), lifestyle behaviours (smoking status, pack-years of smoking, alcohol consumption, reason for quitting smoking or drinking, recreational physical activity, and frequency of using fat to deep fry/pan fry/sauté) medication use, self-rated health status, total energy intake, diabetes, antihypertensive drug use, systolic blood pressure, diastolic blood pressure, dyslipidaemia, waist circumference (as an indicator of adiposity), inclusion of 9 major food groups. |
| Dehghan et al. 2020 (52), 21 countries, Prospective Urban Rural Epidemiology (PURE) study and ONTARGET/TRANSCEND, Prospective Cohort | PURE: Adults from 21 countries  ONTARGET/TRANSCEND: Adults aged ≥ 55 years with a history of coronary, peripheral, or cerebrovascular disease, or diabetes mellitus with end-organ damage  PURE: *n* = 9.5 years  ONTARGET/TRANSCEND *n* = 4.7 years  PURE: *n* = 146011  ONTARGET/TRANSCEND *n* = 31544 | 1 egg = 50g  *n* eggs/week: <1, 1<3, 3<5, 5<7, ≥7  N/A | *PURE study:*  No significant associations for egg intake with CVD mortality, either in total cohort or for those without history of CVD.  *ONTARGET/TRANSCEND studies:*  No significant associations for egg intake with CVD mortality | ↔  ↔ | *PURE*: age; sex; smoking; location; education; physical activity; history of diabetes; daily intakes of fruits, vegetables, dairy, red meat, poultry, and fish; percentage energy from carbohydrate; total daily energy; and centre as a random effect.  *ONTARGET/TRANSCEND*: age; sex; smoking; location; BMI; education; physical activity; history of diabetes; history of myocardial infarction; history of stroke; medication; trial allocation; daily intakes of fruit, vegetables, red meat, poultry, fish, and dairy; and regions as a random effect |
| Huang et al. 2020 (30), United States, National Institutes of Health AARP Diet and Healthy Study, Prospective Cohort | Adults in United States aged 50-71 years  16 years  *n* = 46104 | Egg protein equivalent to 3% energy Intake (g of protein/1000 kcal) | Substitution of plant protein for egg protein equivalent to 3% total energy resulted in reduced risk of CVD mortality for men (HR 0.74 95% CI 0.67-0.82, *P*<0.001) and women (HR 0.72 95% CI 0.63-0.83, *P*<0.001), reduced risk of heart disease for men (HR 0.76 95% CI 0.69-0.85, *P*<0.001) and women (HR 0.72 95% CI 0.62-0.85, *P*<0.001), and reduced risk of stroke in men only (HR 0.67 95% CI 0.52-0.88, *P*=0.003) | ↑ | Age, BMI, alcohol consumption, smoking status, physical activity, race or ethnic group, educational level, marital status, diabetes, health status, vitamin supplement use, daily dietary total energy, animal protein, saturated fat, polyunsaturated fat, monounsaturated fat, trans fat, fibre, vegetables, and fruits, post-menopausal hormone replacement therapy (for women) |
| **Reference, country, data source, study desigb** | **Population characteristics,**  **follow up (years), sample size (n)** | **Egg amount,**  **exposure: categorical, exposure: continuous** | **Study outcomes** | **Risk profile** | **Model for analysis** |
| Pan et al. 2021 (35), United States, Southern Community Cohort Study (SCCS), Shanghai Women's Health Study (SWHS) and the Shanghai Men's Health Study (SMHS), Prospective Cohort | SCCS: Adults from low-income households in United States aged 40-79 years SWHS: Women from Shanghai, China aged 40-70 years SMHS: Men from Shanghai, China aged 40-74 years  12 years  *n* = 202429 | Not specified  *n* eggs/week:  1, 3, 5, 7 & 10  N/A | *White American cohort:*  10 eggs/week v lowest intake: HR 1.14, 95% CI 1.00-1.30 *P*=0.03  *Chinese cohort*:  3 eggs/week v lowest intake: HR 0.95, 95% CI 0.93-0.98, *P*<0.001  5 eggs/week v lowest intake: HR 0.96, 95% CI 0.92-0.99, *P*<0.001  *Black American cohort:*  No significant associations for egg intake with cardiometabolic disease mortality  When separated into cardiometabolic subtypes (CHD, stroke or diabetes), significance was lost for all cohorts  (*cardiometabolic = CHD, stroke, or diabetes*) | ↑White American    ↓Chinese    ↔ Black American  ↔ All | Age, sex, education, annual income, marital status, total energy intake, smoking status, alcohol consumption, physical activity level, body mass index, healthy eating index, history of diabetes, hypertension, dyslipidaemia, coronary heart disease, stroke and hormone replacement therapy (for women) |
| Ruggiero et al. 2021 (46), Italy, Moli-sani Study, Prospective Cohort | Adults in Italy aged ≥35 years  8.2 years  *n* = 20562 | 1 egg = 50g  *n* eggs/week: ≤1, >1≤2, >2≤4 & >4  1 egg/week increment | Highest intake v lowest intake: HR 1.75, 95% CI 1.07-2.87, *P*trend=0.010  Second highest intake v lowest intake: HR 1.43, 95% CI 1.03-1.97, Ptrend=0.010  Continuous per 1 egg/week: HR 1.10, 95% CI 1.01-1.21 *P*=0.036 | ↑ | Age, sex, energy intake, educational level, household income, residence, smoking, BMI, leisure-time PA , baseline diabetes, hypertension , hyperlipidaemia, Mediterranean diet score |
| Sun et al. 2021 (29), United States, Women's Health Initiative, Prospective Cohort | Post-menopausal women in United States aged 50-79 years  18.1 years  *n* = 102521 | Not specified  median intake, *n* eggs/day: 0.03, 0.1, 0.2, 0.3 & 0.7  1 egg/day increment | Highest intake v lowest intake: HR 1.24, 95% CI 1.14 1.34, *P*trend<0.001  Continuous per 1 egg/day: HR 1.21, 95% CI 1.14-1.28 | ↑ | Age at baseline, race/ethnicity, education, income, observational study/clinical trials, unopposed estrogen use, estrogen/progesterone use, smoking status, physical activity, alcohol intake, total energy intake, baseline diabetes mellitus status, baseline high blood cholesterol status, family history of heart attack/stroke, wholegrain consumption, vegetable consumption, fruit consumption, sugar-sweetened beverage consumption, and mutual adjustment for other protein sources |
| Xia et al. 2020 (27), United States, National Health and Nutrition Examination Survey 1999-2014, Prospective Cohort | Adults in United States aged ≥20 years  7.8 years  *n* = 37121 | Not specified  *n* eggs/day): <0.5, 0.5-1, >1  0.5 egg/day increment | No significant association with heart disease mortality for highest intake v lowest intake, or per 0.5 egg/day continuous | ↔ | Age, sex, race/ethnicity, education, family income-poverty ratio, marital status, and National Health and Nutrition Examination Survey cycles, total energy intake, cigarette smoking, alcohol drinking, physical activity, BMI, baseline hypertension, diabetes mellitus, hypercholesterolemia, cardiovascular disease, cancer |

| **Reference, country, data source, study design** | **Population characteristics,**  **follow up (years), sample size (n)** | **Egg amount,**  **exposure: categorical, exposure: continuous** | **Study outcomes** | **Risk profile** | **Model for analysis** |
| --- | --- | --- | --- | --- | --- |
| Xu et al. 2019 (15), China, Guangzhou Biobank Cohort Study, Prospective Cohort | Adults in Guangzhou, China aged ≥50 years  9.8 years  *n* = 28024 | Not specified  *n* eggs/week: <1, 1-2, 3-4, 5-6, & 7+  N/A | No significant association for highest intake v lowest intake, including CVD mortality subtypes (IHD, total stroke, ischemic stroke, haemorrhagic stroke, combined IHD and ischemic stroke) | ↔ | Sex, age, education, occupation, family income, smoking status, physical activity, alcohol drinking, self-rated health and chronic disease history (diabetes, hypertension and dyslipidaemia) |
| Zamora-Ros et al. 2019 (44), Spain, European Prospective Investigation into Cancer and Nutrition, Prospective Cohort | Adults in Spain aged 29-69 years  18 years  *n* = 40621 | 1 egg = 50g  g/day: <10.8, 10.8-19.9, 20.0-30.4 & >30.4  1 egg/week increment | No significant associations between egg consumption (highest v lowest intake or 1 egg/week continuous), including CVD mortality subtypes (ischaemic heart diseases and cerebrovascular diseases) | ↔ | Age at recruitment, sex, smoking intensity, BMI, lifetime alcohol intake, education level, physical activity, energy intake, adherence to Mediterranean diet, centre for recruitment |
| Zhuang et al. 2021 (24), United States, National Institutes of Health AARP Diet and Healthy Study, Prospective Cohort | Adults in United States aged 50-71 years  16 years  *n* = 521120 | Not specified  median intake, egg g/2000kcal/day: 0, 3.0, 7.5, 13.7 & 28.7  0.5 egg/day increment | Highest intake v lowest intake: HR 1.14, 95% CI 1.11-1.18, *P*trend<0.001  Continuous per 0.5 egg/day: HR 1.07, 95% CI 1.06-1.09  Highest intake v lowest intake adjusted for dietary cholesterol:  HR 1.01, 95% CI 0.96-1.06, *P*trend=0.11    No significant association for consumption of egg whites or substitutes | whole eggs ↑    adjusted for dietary cholesterol ↔  egg whites ↔ | Age, sex, body mass index, race, education, marital status, household income, smoking status, alcohol consumption, physical activity, usual activity at work, history of hypertension, cholesterol level, heart disease, stroke, diabetes or cancer at baseline, total energy, egg whites/substitutes, Healthy Eating Index 2015 |
| Zupo et al. 2020 (43), Italy, Multi-center Italian study on Cholelithiasis, Prospective Cohort | Adults in Italy aged  ≥30 years  34 years  *n* = 2472 | Not specified  Frequency:  0 = never, 1 = rarely, 2 = occasionally, 3 = frequently, 4 = daily  N/A | Reduced risk of CVD and stroke mortality with high frequency egg consumption: HR 0.73, 95% CI 0.14-1.45 | ↓ | Sex, age, BMI, education, smoking, comorbidity, wine and olive oil |

| **Reference, country, data source, study design** | **Population characteristics,**  **follow up (years), sample size (n)** | **Egg amount,**  **exposure: categorical, exposure: continuous** | **Study outcomes** | **Risk profile** | **Model for analysis** |
| --- | --- | --- | --- | --- | --- |
| **Egg Consumption and CVD Incidence** | | | | | |
| Abdollahi et al. 2019 (49), Finland, Kuopio Ischaemic Heart Disease Risk Factor Study, Prospective Cohort | Males in Kuopio, Finland and surrounding rural communities aged 42-60 years  21.2 ± 7.2 years  *n* = 1950 | 1 egg = 55g  *n* grams/day: <15, 15-26, 27-45, >45  1 egg/week increment | No significant association for egg intake with total stroke, ischemic stroke or haemorrhagic stroke, either by comparison of highest intake v lowest intake, or per 1 egg/week continuous | ↔ total stroke  ↔ ischemic stroke  ↔ haemorrhagic stroke | Age, examination year, and energy intake, BMI (kg/m2), pack-years of smoking, leisure-time physical activity, hypertension medication, and intakes of alcohol and fruit, berries, and vegetables |
| Al-Ramady et al. 2022 (31), United States, Million Veteran Program, Prospective Cohort | Veterans in United States aged 65.5±11.7 years (91.6% men) with no history of stroke  3.3 years  *n* = 233792 | Not specified  *n* eggs/*time*: <1/month, 1-3/month, 1/week, 2-4/week, 5-6/week, 1/day, ≥2/day  N/A | *For ischemic stroke:*  Highest v lowest intake: HR 1.22, 95% CI 1.03-1.45  Second highest v lowest intake: HR 1.20, 95% CI 1.03-1.40  Third highest v lowest intake: HR 1.16, 95% CI 1.01-1.33  (*P*trend=0.0085 for all)  *For haemorrhagic stroke:*  No significant association for egg intake with haemorrhagic stroke | ↑ischemic stroke  ↔ haemorrhagic stroke | Age, gender, race, BMI, DASH score, education status |
| Chen et al. 2021 (34), United States, Women’s Health Initiative, Prospective Cohort | Post-menopausal women in United States aged 50-79 years  17.8 years  *n* = 96831 | 1 egg = 1 large whole egg  *n* eggs/week: <1, 1<2, 2<3, 3<7, ≥7  0.5 egg/day increment | *For CVD incidence:*  Highest intake v lowest intake: HR 1.14, 95% CI 1.04-1.25, *P*trend=0.004  *For ischemic heart disease:*  Highest intake v lowest intake: HR 1.11, 95% CI 1.00-1.23, *P*trend=0.040  *For ischemic stroke:*  Highest intake v lowest intake: 1.40, 95% CI 1.18-1.66, *P*trend=0.001  *For haemorrhagic stroke:*  No significant association for egg intake with haemorrhagic stroke | ↑CVD incidence  ↑IHD  ↑ischemic stroke  ↔ haemorrhagic stroke | Age, region, ethnicity, socioeconomic status (education, family income, health insurance), lifestyle behaviours (smoking status, pack-years of smoking, alcohol consumption, reason for quitting smoking or drinking, recreational physical activity, and frequency of using fat to deep fry/pan fry/sauté) medication use, self-rated health status, total energy intake, diabetes, antihypertensive drug use, systolic blood pressure, diastolic blood pressure, dyslipidaemia, waist circumference (as an indicator of adiposity), inclusion of 9 major food groups. |

| **Reference, country, data source, study design** | **Population characteristics,**  **follow up (years), sample size (n)** | **Egg amount,**  **exposure: categorical, exposure: continuous** | **Study outcomes** | **Risk profile** | **Model for analysis** |
| --- | --- | --- | --- | --- | --- |
| Dehghan et al. 2020 (52), 21 countries,  Prospective Urban Rural Epidemiology (PURE) and ONTARGET/TRANSCEND, Prospective Cohort | PURE: Adults from 21 countries  ONTARGET/TRANSCEND: Adults aged ≥ 55 years with a history of coronary, peripheral, or cerebrovascular disease, or diabetes mellitus with end-organ damage  PURE: 9.5 years  ONTARGET/TRANSCEND: 4.7 years  PURE: *n* = 146011  ONTARGET/TRANSCEND *n* = 31544 | 1 egg = 50g  *n* eggs/week: <1, 1<3, 3<5, 5<7, ≥7  N/A | PURE study data*:*  *For major CVD events*:  Highest v lowest intake: HR 0.89, 95% CI 0.82-0.97, *P*trend=0.04  After excluding those with a history of CVD, this result was no longer significant  *For MI:*  Highest intake v lowest intake: HR 0.83, 95% CI 0.72-0.95, *P*trend=0.004 (entire cohort) and HR 0.84, 95% CI 0.72-0.98, *P*trend=0.02 (those without history of CVD)  *For stroke and heart failure:*  No significant association for egg intake with stroke or heart failure  ONTARGET/TRANSCEND study data:  No significant associations for egg intake with major CVD events, myocardial infarction, stroke or heart failure | ↓major CVD (PURE, whole cohort)  ↔ major CVD (PURE, no history of CVD)  ↓MI  (PURE, whole cohort)  ↓MI  (PURE, no history of CVD)  ↔ stroke, heart failure (PURE)  ↔ major CVD, MI, stroke, heart failure (ONTARGET/TRANSCEND) | *PURE*: age; sex; smoking; location; education; physical activity; history of diabetes; daily intakes of fruits, vegetables, dairy, red meat, poultry, and fish; percentage energy from carbohydrate; total daily energy; and centre as a random effect.  *ONTARGET/TRANSCEND*: age; sex; smoking; location; BMI; education; physical activity; history of diabetes; history of myocardial infarction; history of stroke; medication; trial allocation; daily intakes of fruit, vegetables, red meat, poultry, fish, and dairy; and regions as a random effect |
| Djousse et al. 2020 (32), United States, Million Veteran Program, Prospective Cohort | Veterans in United States aged 64.4±12 years (90.1% men) with no history of CAD  3.24 years  *n* = 188267 | Not specified  *n* eggs/*time*: <1/month, 1-3/month, 1/week, 2-4/week, 5-6/week, 1/day, ≥2/day  N/A | *For MI:*  Highest intake v lowest intake: HR 1.13, 95% CI 1.00-1.28,  Second highest intake v lowest intake: HR 1.11, 95% CI 1.00-1.24 *(P*non-linear=0.019 for both) | ↑MI | Age, sex, race, education, BMI,  exercise, smoking, alcohol intake, and DASH score |

| **Reference, country, data source, study design** | **Population characteristics,**  **follow up (years), sample size (n)** | **Egg amount,**  **exposure: categorical, exposure: continuous** | **Study outcomes** | **Risk profile** | **Model for analysis** |
| --- | --- | --- | --- | --- | --- |
| Djousse et al. 2021 (33), United States, Atherosclerosis Risk in Communities, Cardiovascular Health, Coronary Artery Risk Development in Young Adults, Jackson Heart Study, Multi-Ethnic Study of Atherosclerosis, Physicians' Health, Reasons for Geographic and Racial Differences in Stroke, Women's Antioxidant Cardiovascular Study, Women's Health Study, Prospective Cohort | Adults in United States aged 25 to 72 years  7-20.8 years  *n* = 103811 | Not specified  *n* eggs/*time*: <1/month, 1-3/month, 1/week, 2-4/week, 5-6/week, ≥7 eggs/week  N/A | No significant association with egg intake and CHD in pooled analysis  In subgroup analysis of older cohort (60+ years), second highest intake v lowest intake: HR 1.30, 95% CI 1.03-1.56 (not significant for highest v lowest intake) | ↔CHD  ↑CHD (older cohort, 5-6 eggs/week only) | Age, field centre, body mass index, smoking, alcohol intake, education, physical activity, dyslipidaemia and DASH score |
| Key et al. 2019 (48), 9 European countries, European Prospective Investigation into Cancer and Nutrition, Prospective Cohort | Adults in 9 European countries without reported or known history of MI or stroke at baseline  12.6 years  *n* = 409885 | Not specified  *n* grams/day: 8, 12, 14, 18 & 26  20g egg/day increment | *For IHD:*  Continuous per 20g egg/day: HR 0.93, 95% CI 0.88-0.99, *P*trend=0.023 (after excluding first 4 years of follow up this was no longer significant)  No significant association found in categorical analysis  In substitution analysis, substitution of 100 kcal/day from red or processed meat with equivalent eggs: HR 0.76, 95% CI 0.62-0.92 | ↓IHD | Age, smoking status and number of cigarettes per day, history of diabetes mellitus, previous hypertension, prior hyperlipidaemia, Cambridge physical activity index, employment status, level of education completed, BMI, current alcohol consumption, and observed intakes of energy, fruit and vegetables combined, sugars (as percent energy), and fibre from cereals, and stratified by sex and EPIC centre where data was collected |
| Kunutsor et al. 2022 (50), Finland, Kuopio Ischaemic Heart Disease Risk Factor Study, Prospective Cohort | Men in Kuopio, Finland aged 42-61 years without history of VTE or CHD  23.8 years  *n* = 1852 | Not specified  *n* grams/day: <20, 20-38, >38  55g/day increment | No significant association for egg intake with VTE for highest v lowest intake, or per 55g/day continuous | ↔ VTE | Age, energy intake, systolic BP, BMI, TAG, smoking status, alcohol consumption, physical activity, socio-economic status, serum albumin, intake of fruits, berries and vegetables, intake of processed and unprocessed red meat and history of cancer, history of T2D, serum cholesterol, serum TAG, serum high-sensitivity C-reactive protein |
| Mohammadifard et al. 2022 (51), Iran, Isfahan cohort study, Prospective Cohort | Adults in Iran aged 35+ years with no history of CVD at baseline  11.3 years  *n* = 4367 | Not specified  Frequency of consumption: times per week: <1, 1-2, 2+  N/A | No significant association for egg intake CVD, myocardial infarction, stroke or coronary heart disease | ↔ CVD  ↔ MI  ↔ stroke  ↔ CHD | Age, sex, education, residency (urban/rural), smoking status, daily physical activity, family history of CVD, metS, aspirin, BMI, Global Dietary Index |
| Tong et al. 2020 (45), 9 European countries, European Prospective Investigation into Cancer and Nutrition, Prospective Cohort | Adults in 9 European countries (excluding France), without known or possible history of stroke or MI  12.7 years  *n* = 418329 | Not specified  N/A  20g/day increment | *For total stroke:*  Continuous per 20 g/day: HR 1.07, 95% CI 1.01-1.14, *P*trend=0.031  *For ischemic stroke:*  No significant association for egg intake with ischemic stroke  *For haemorrhagic stroke:*  Continuous per 20g/day: HR 1.25, 95% CI 1.09-1.43, *P*trend=0.002 | ↑ total stroke  ↔ ischemic stroke  ↑ haemorrhagic stroke | Age, smoking status and number of cigarettes per day, self-reported history of diabetes, hypertension, or hyperlipidaemia, Cambridge physical activity index, employment status, level of education completed, current alcohol consumption, body mass index, and calibrated or observed intake of energy, as appropriate, and stratified by sex and EPIC centre |
| Wang et al. 2022 (28), United States, Cardiovascular Health Study, Prospective Cohort | Adults in United States aged 65+ years without prevalent CVD  12.5 years  *n* = 3931 | Not specified  N/A  Per IQR 0.47 serving/day | No significant association for egg intake with atherosclerotic CVD | ↔ atherosclerotic CVD | Age, sex, race, study site, education, income, and time-varying self-reported health status, smoking status, alcohol intake, physical activity, antibiotic use, and intakes of total energy, fruits, vegetables, dietary fibre, total dairy products, and the other animal source foods mutually adjusted. |
| Wang et al. 2022 (39), China, China Family Panel Studies, Prospective Cohort | Adults in China aged 16-110 years without chronic disease  6 years  *n* = 20688 | Not specified  Frequency of consumption: times per week: 0, 1-2, 3-6, 7+  N/A | *For cardiovascular disease:*  Highest intake v lowest intake: HR 0.83, 95% CI: 0.72-0.95  Second highest intake v lowest intake: HR 0.78, 95% CI: 0.69-0.88  Third highest intake v lowest intake: HR 0.84, 95% CI: 0.74-0.94  (*P*trend=0.002 for all) | ↓ CVD | Gender, age, BMI, ethnicity, marital status, residential region, education attainment, employment status, annual household income, physical activity, smoking status, alcohol consumption, sleep duration, meat intake, vegetable intake, egg. |

| **Reference, country, data source, study design** | **Population characteristics,**  **follow up (years), sample size (n)** | **Egg amount,**  **exposure: categorical, exposure: continuous** | **Study outcomes** | **Risk profile** | **Model for analysis** |
| --- | --- | --- | --- | --- | --- |
| Xia et al. 2020 (38), China, Prediction for Atherosclerotic Cardiovascular Disease Risk in China, Prospective Cohort | Adults in China free of CVD  7.6 years  *n* = 102136 | Not specified  *n* eggs/week: <1, 1<3, 3<6 (reference), 6<10, ≥10  N/A | *For cardiovascular disease:*  Highest intake v reference: HR 1.39, 95% CI: 1.28-1.52  Second highest intake v reference: HR 1.25, 95% CI 1.14-1.38  Lowest intake v reference: HR 1.22, 95% CI: 1.11-1.35  Second lowest intake v reference: HR 1.09, 95% CI 1.00-1.19  *For coronary heart disease:*  Highest intake v reference: HR 1.86, 95% CI: 1.57-2.22  Second highest intake v reference: HR 1.34, 95% CI: 1.12-1.61  No significant associations for low intakes  *For total stroke:*  Highest intake v reference: HR 1.18, 95% CI: 1.05-1.33  Second highest intake v reference: HR 1.21, 95% CI 1.07-1.36  Lowest intake v reference: HR 1.27, 95% CI: 1.12-1.44  Second lowest intake v reference: HR 1.13, 95% CI 1.01-1.26  *For ischemic stroke:*  Highest intake v reference: HR 1.33, 95% CI: 1.15-1.54  Second highest intake v reference: HR 1.28, 95% CI 1.10-1.49  Lowest intake v reference: HR 1.20, 95% CI: 1.02-1.42  *For haemorrhagic stroke:*  Lowest intake v reference: HR 1.34, 95% CI: 1.07-1.68  Second lowest intake v reference: HR 1.23, 95% CI 1.01-1.50  No significant association for high intakes | ↑CVD incidence (high and low intake)  ↑CHD (high intake only)  ↑total stroke (high and low intake)  ↑ischemic stroke (high and low intake)  ↑haemorrhagic stroke (low intake only) | Age, gender, urban or rural resident, per-capita household income, education attainment, tobacco smoking, alcohol consumption and family history of CVD, physical activity, BMI and dietary factors (red meat intake, fresh fruit and vegetable intake) |
| Zhong et al.2019 (25), United States, Atherosclerosis Risk in Communities Study, Coronary Artery Risk Development in Young Adults Study, Framingham Heart Study, Framingham Offspring Study, Jackson Heart Study, and the Multi-Ethnic Study of Atherosclerosis, Prospective Cohort | Adults in United States without CVD  17.5 years  *n* = 29615 | Not stated  N/A  0.5 egg/day increment | *For CVD:*  Continuous per 0.5 egg/day: HR 1.06, 95% CI 1.03-1.20  (no longer significant after adjusting for cholesterol intake but remained significant when adjusting for saturated fat, unsaturated fat and trans fat intakes) | ↑CVD | Age, sex, race/ethnicity, education, total energy, smoking status, smoking pack-years, cohort-specific physical activity z score, alcohol intake, use of hormone therapy  Secondary adjustment: cholesterol, saturated fat, unsaturated fat and trans fat. |

| **Reference, country, data source, study design** | **Population characteristics,**  **follow up (years), sample size (n)** | **Egg amount,**  **exposure: categorical, exposure: continuous** | **Study outcomes** | **Risk profile** | **Model for analysis** |
| --- | --- | --- | --- | --- | --- |
| Zhong et al. 2021 (26), United States, Atherosclerosis Risk in Communities Study, Coronary Artery Risk Development in Young Adults Study, Cardiovascular Health Study, Framingham Heart Study, Framingham Offspring Study, and the Multi-Ethnic Study of Atherosclerosis, Prospective Cohort | Adults in United States without CVD  19 years  *n* = 29682 | 1 serving = 1 egg | Substituting eggs with fish, nuts, legumes or whole grains was associated with 2–3% lower relative risks for incident CVD when the substitution amount was one serving per week, and 15–21% lower relative risks when the substitution amount was one serving per day. | ↑CVD | Age, sex, race and ethnicity, education, total energy, smoking status, smoking pack-years, cohort-specific physical activity z score, alcohol intake, hormone therapy and Alternative Healthy Eating Index 2010 (excluding alcohol, meats, nuts, legumes and whole grains). |
| **Egg Consumption and CVD Risk Factors** | | | | | |
| Abdollahi et al. 2019 (49), Finland, Kuopio Ischaemic Heart Disease Risk Factor Study, Prospective Cohort | Males in Kuopio, Finland and surrounding rural communities aged 42-60 years  21.2 ± 7.2 years  *n* = 1950 | 1 egg = 55g  *n* grams/day: <15, 15-26, 27-45, >45  1 egg/week increment | *For diastolic blood pressure:*  Highest intake v lowest intake: MD -1.6 mmHg, *P*trend=0.04  No associations with systolic blood pressure. | DBP↓  SBP↔ | Age, examination year, energy intake, BMI, pack-years of smoking, leisure-time physical activity, hypertension medication, and intakes of alcohol and fruit, berries, and vegetables |
| Dehghan et al. 2020 (52), 21 countries,  Prospective Urban Rural Epidemiology (PURE) study and ONTARGET/TRANSCEND, Prospective Cohort | PURE: Adults in 21 countries  ONTARGET/TRANSCEND: Adults aged ≥ 55 years with a history of coronary, peripheral, or cerebrovascular disease, or diabetes mellitus with end-organ damage  PURE: 9.5 years  ONTARGET/TRANSCEND: 4.7 years  PURE: *n* = 146011  ONTARGET/TRANSCEND: *n* = 31544 | 1 egg = 50g  *n* eggs/week: <1, 1<3, 3<5, 5<7, ≥7  N/A | *For diastolic blood pressure:*  Highest intake v lowest intake: MD -1.0 mmHg, *P*trend<0.001 (PURE)  Highest intake v lowest intake: MD 1.5 mmHg, *P*trend=0.04 (ONTARGET/TRANSCEND)  *For systolic blood pressure:*  Highest intake v lowest intake: MD -1.3 mmHg, *P*trend<0.001 (PURE)  Highest intake v lowest intake: MD 2.3 mmHg, *P*trend=0.02 (ONTARGET/TRANSCEND)  No significant association for total cholesterol, LDL-C, HDL-C, TC: HDL-C ratio or TAG | DBP↓(PURE)  DBP↑(ONTARGET/TRANSCEND)  SBP↓(PURE)  SBP↑(ONTARGET/TRANSCEND)  Lipids↔ | Age, sex, education, urban or rural location, smoking, physical activity, history of diabetes, fruit and vegetables, red meat, poultry, fish, dairy, percentage of energy from carbohydrates, and total energy intake. |

| **Reference, country, data source** | **Population characteristics,**  **follow up (years), sample size (n)** | **Egg amount,**  **exposure: categorical, exposure: continuous** | **Study outcomes** | **Risk profile** | **Model for analysis** |
| --- | --- | --- | --- | --- | --- |
| Liang et al. 2021 (42), China, China Health and Nutrition Survey, Prospective Cohort | Adults in China aged 18-60 years  6.81 years  *n* = 5394 | 1 serving = 50g  Substitution analysis: 1 serving/day of red or processed meat with 1 serving/day eggs | Substituting eggs for red or processed meat was associated with a lower risk of hypertension.  Replacing 1 serving/day of red or processed meat (serving not specified) with 1 serving/day (50g) of eggs was associated with a 14.3% lower risk of hypertension (HR 0.857, 95% CI 0.784-0.936) | Hypertension↓ | Age, sex, BMI, marital status, urban location, education degree, working status, smoking, alcohol consumption, time spent performing physical activity, time spent sedentary, and time spent in bed, consumption of vegetables, fruit, nuts and legumes, whole grains, sweetened beverages, sodium intake, and total energy intake. |
| Liu et al. 2020 (41), China, Healthy Survey in Hanzhong rural area of Shanxi province in China, Cross-sectional Cohort | Adults in rural China aged 18-80 years  N/A (baseline data only)  *n* = 2241 | N/A  *n* grams/day: 0, <10, 10, 20, 40, 50 (all categories approximate)  10g/day increment | *For females:*  *Central obesity by WC:*  Highest intake v lowest intake: OR 0.66, 95% CI: 0.39-0.99, *P*trend=0.034  *Excessive body fat by BF%:*  Highest intake v lowest intake: OR 0.62, 95% CI: 0.37-1.00, *P*trend=0.005  *Obesity by BMI:*  No significant association  *Normal weight with excessive BF%*  Continuous per 10g/day: OR 0.93, 95% CI: 0.87-1.00, *P*trend=0.048  *Normal weight with central obesity*  No significant association  *Overweight with excessive BF%*  Continuous per 10g/day: OR 0.87, 95% CI: 0.80-0.93, *P*trend<0.001  *Overweight with central obesity*  Continuous per 10g/day: OR 0.87, 95% CI: 0.80-0.94, *P*trend=0.001  *Males:* No significant associations found for males | Overweight/ obesity↓ (females)  Males ↔ | Energy, age, education, fortune index, physical activity level, alcohol intake, smoking status, red meat, fruit and vegetable intake and fat intake. |
| Macdonald et al. 2020 (47), France, Etude Epidémiologique de femmes de la Mutuelle Générale de l0Education, Prospective Cohort | Women in France aged 40-65  19.1 years  *n* = 46501 | 1 egg = 60g  *n* eggs/week: <1, 1-1.9, 2-2.9, 3-3.9, 4-6.9, ≥7  N/A | *For hypertension*  Second highest v lowest intake: HR 1.14, 95% CI: 1.06-1.18  Third highest v lowest intake: HR 1.10, 95% CI: 1.04-1.17  Third lowest v lowest intake: HR 1.07, 95% CI: 1.01-1.13  (*P*trend<0.005 for all)  No significant association for highest intake v lowest intake with risk of hypertension | Hypertension↑ (mid-range intakes)  Hypertension ↔ (high intake) | Total physical activity, total calories, smoking, family history of CVD, education level, menopausal status, use of MHT, dyslipidaemia, and diabetes; Mediterranean diet score. |

| **Reference, country, data source, study design** | **Population characteristics,**  **follow up (years), sample size (n)** | **Egg amount,**  **exposure: categorical, exposure: continuous** | **Study outcomes** | **Risk profile** | **Model for analysis** |
| --- | --- | --- | --- | --- | --- |
| Pan et al. 2021 (40), China, China Kadoorie Biobank, Nested Case-Control | Adults in urban and rural areas in China aged 30-79 years  N/A (baseline data only)  *n* = 4778 | Not specified  Frequency of consumption: days per week: 0, 0.5, 2, 5, 7  N/A | Egg consumption was positively associated with lipoprotein particle concentrations of very large and large HDL (*P*<0.01). Similarly, within very large and large HDL, there were positive associations of total lipids (*P*<0.01), total cholesterol including its subclasses (cholesterol esters and free cholesterol), or phospholipids with egg consumption. Conversely, there was an inverse association of cholesterol esters in small VLDL with egg consumption (*P*<0.05). In addition to the absolute concentrations of lipids, the percentage of total cholesterol and cholesterol esters in large HDL were positively associated with egg consumption (*P*<0.05). | ↓ | Age, sex, region, education, household income, occupation, marital status, tea-drinking habit, smoking status, alcohol intake, physical activity, self-rated health, fasting time, and frequency of other 11 food groups. |
| Riseberg et al. 2022 (36), United States, Boston Puerto Rican Health Study, Prospective Cohort | Puerto Ricans residing in Boston Massachusetts United States or surrounding area aged 45-75 years  2 years  *n* = 1126 | Not specified  N/A  1 serving/day increment | No significant associations found with egg consumption and HDL-C, TAG, systolic and diastolic blood pressure or WC | ↔ | Energy intake, sex, age, education, baseline outcome, smoking, alcohol intake, physical activity, psychological acculturation, fruit and vegetable intake score, omega-3 fatty acid intake, whole grain intake, medication (for blood pressure, triglycerides, and glucose only), and sodium intake (for blood pressure only). |
| Xu et al. 2019 (15), China, Guangzhou Biobank Cohort Study, Prospective Cohort | Adults in China aged ≥50 years  9.8 years  *n* = 28024 | Not specified  *n* eggs/week: <1, 1-2, 3-4, 5-6, ≥7  N/A | *For systolic blood pressure (*mmHg)*:*  Highest intake v lowest intake: MD -3.32mmHg, 95% CI: -4.6, -2.04, *P*trend<0.001  *For diastolic blood pressure (*mmHg)*:*  Highest intake v lowest intake: MD -1.50, 95% CI: -2.18, -0.82, *P*trend<0.001  *For LDL-C (mmol/L):*  Highest intake v lowest intake: MD -0.07, 95% CI: -0.11, -0.02, *P*trend<0.001  *For HDL-C (mmol/L):*  No significant associations found with egg consumption and HDL-C  *For TAG (mmol/L):*  Highest intake v lowest intake: MD -0.14, 95% CI: -0.21, -0.06, *P*trend<0.001  *For TC (mmol/L):*  Highest intake v lowest intake: MD -0.10, 95% CI: -0.17, -0.03, *P*trend=0.001  *For BMI (kg/m^2^):*  Highest intake v lowest intake: MD -0.29, 95% CI: -0.50, -0.09, *P*trend=0.006  *For WC (cm):*  No significant associations found with egg consumption and WC | SBP↓  DBP↓  LDL-C↓  HDL-C↔  TAG↓  TC↓  BMI↓  WC↔ | Sex, age, education, occupation, family income, smoking status, physical activity, alcohol drinking, self-rated health, chronic disease history (diabetes, hypertension and dyslipidaemia) and dietary foods and energy intake |
| **Reference, country, data source, study design** | **Population characteristics,**  **follow up (years), sample size (n)** | **Egg amount,**  **exposure: categorical, exposure: continuous** | **Study outcomes** | **Risk profile** | **Model for analysis** |
| Zhang et al.2022 (37), China, Prediction for Atherosclerotic Cardiovascular Disease Risk in China, Longitudinal | Adults in China  N/A (baseline data only)  *n* = 60952 | 1 egg = 50g  *n* eggs/week: <3, 3<6, ≥6  N/A | *For TC (mg/dL):*  Highest intake v lowest intake: MD 1.795, 95% CI: 1.315 to 2.275  Moderate intake v lowest intake: MD -0.606, 95% CI: -1.129 to -0.084  *For LDL-C (mg/dL):*  Highest intake v lowest intake: MD 1.763, 95% CI: 1.33 to 2.189  Moderate intake v lowest intake: MD -0.848, 95% CI: -1.318 to -0.377  *For non-HDL-C (mg/dL):*  Highest intake v lowest intake: MD 0.917, 95% CI: 0.452 to 1.381  Moderate intake v lowest intake: MD -1.071, 95% CI: -1.581 to -0.561  *For TAG (mg/dL):*  Highest intake v lowest intake: MD -4.208, 95% CI: -5.485 to -2.931  Moderate intake v lowest intake: MD -1.465, 95% CI: -2.852 to -0.079  *For HDL-C (mg/dL):*  Highest intake v lowest intake: MD 0.815, 95% CI: 0.612 to 1.018  Moderate intake v lowest intake: MD 0.461, 95% CI: 0.240 to 0.682 | TC↑ (high intake)  TC↓ (moderate intake)  LDL-C↑ (high intake)  LDL-C↓ (moderate intake)  Non-HDL-C↑ (high intake)  Non-HDL-C↓ (moderate intake)  TG↓ (high and moderate intake)  HDL-C↑ (high and moderate intake) | Age, gender, urbanization, geographic region, body mass index, current smoker, current drinker, education level, per-capita household income, ideal diet score, and ideal dairy intake |

Abbreviations: AARP, American Association of Retired Persons; BF, body fat; BMI, body mass index; CAD, coronary artery disease; CHD, coronary heart disease; CI, confidence interval; CVD, cardiovascular disease; DASH, dietary approach to stop hypertension; DBP, diastolic blood pressure; g, grams; HDL-C, high density lipoprotein cholesterol; HR, hazard ratio; IHD, ischemic heart disease; IQR, interquartile range; kcal, kilocalories; LDL-C, low density lipoprotein cholesterol; MD, mean difference; metS, metabolic syndrome; MHT, menopause hormone therapy; MI, myocardial infarction; n, number; N/A, not applicable; OR, odds ratio; SBP, systolic blood pressure; T2D, type 2 diabetes; TAG; triacylglycerides; TC, total cholesterol; VLDL, very low-density lipoprotein cholesterol; VTE, venous thromboembolism; WC, waist circumference; ↑significantly increased risk; ↓, significantly decreased risk; ↔, no significant association
